# 1-Methyl-3,5-bis­[(*E*)-(3-methyl-2-thienyl)methyl­ene]piperidin-4-one monohydrate

**DOI:** 10.1107/S1600536809010393

**Published:** 2009-03-28

**Authors:** K. Rajeswari, K. Pandiarajan, P. Gayathri, A. Thiruvalluvar

**Affiliations:** a Department of Chemistry, Annamalai University, Annamalai Nagar 608 002, Tamilnadu, India; b PG Research Department of Physics, Rajah Serfoji Government College (Autonomous), Thanjavur 613 005, Tamil Nadu, India

## Abstract

In the title mol­ecule, C_18_H_19_NOS_2_·H_2_O, the piperidine ring adopts an envelope conformation with the methyl substituent in an equatorial position. Each of the olefinic double bonds has an *E* configuration. The dihedral angle between the two thio­phene rings is 6.04 (14)°. The water mol­ecule forms two donor inter­actions, one with the carbonyl O atom and the other to the hetero N atom. The centrosymmetric {C_18_H_19_NOS_2_·H_2_O}_2_ pairs thus formed are linked into a supra­molecular chain *via* C—H⋯O_water_ contacts.

## Related literature

For piperidine-4-ones as anti­mycobacterial agents, see: Jha & Dimmock (2006 ▶). For their cytotoxic properties, see: Das *et al.* (2007 ▶).
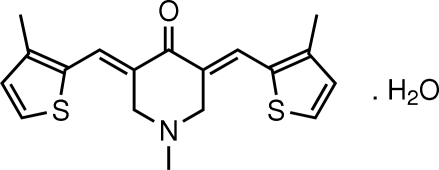


## Experimental

### 

#### Crystal data

C_18_H_19_NOS_2_·H_2_O
*M* *_r_* = 347.50
Triclinic, 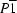

*a* = 7.5781 (7) Å
*b* = 10.9926 (9) Å
*c* = 11.5304 (10) Å
α = 79.531 (2)°
β = 83.404 (2)°
γ = 71.673 (2)°
*V* = 894.90 (14) Å^3^
*Z* = 2
Mo *K*α radiation
μ = 0.31 mm^−1^
*T* = 293 K
0.36 × 0.22 × 0.22 mm

#### Data collection

Bruker Kappa APEXII CCD diffractometer
Absorption correction: multi-scan (*SADABS*; Bruker, 2004 ▶) *T*_min_ = 0.901, *T*_max_ = 0.938
15827 measured reflections
3127 independent reflections
2686 reflections with *I* > 2σ(*I*)
*R*_int_ = 0.021

#### Refinement

*R*[*F* ^2^ > 2σ(*F* ^2^)] = 0.043
*wR*(*F* ^2^) = 0.130
*S* = 1.11
3127 reflections
217 parameters
3 restraints
H atoms treated by a mixture of independent and constrained refinement
Δρ_max_ = 0.44 e Å^−3^
Δρ_min_ = −0.31 e Å^−3^

### 

Data collection: *APEX2* (Bruker, 2004 ▶); cell refinement: *SAINT-NT* (Bruker, 2004 ▶); data reduction: *SAINT-NT*; program(s) used to solve structure: *SHELXS97* (Sheldrick, 2008 ▶); program(s) used to refine structure: *SHELXL97* (Sheldrick, 2008 ▶); molecular graphics: *ORTEP-3* (Farrugia, 1997 ▶); software used to prepare material for publication: *PLATON* (Spek, 2009 ▶).

## Supplementary Material

Crystal structure: contains datablocks global, I. DOI: 10.1107/S1600536809010393/tk2393sup1.cif

Structure factors: contains datablocks I. DOI: 10.1107/S1600536809010393/tk2393Isup2.hkl

Additional supplementary materials:  crystallographic information; 3D view; checkCIF report

## Figures and Tables

**Table 1:** Hydrogen-bond geometry (Å, °)

*D*—H⋯*A*	*D*—H	H⋯*A*	*D*⋯*A*	*D*—H⋯*A*
O1*W*—H1*A*⋯N1	0.86 (4)	2.03 (4)	2.867 (4)	164 (4)
O1*W*—H1*B*⋯O4^i^	0.86 (4)	1.91 (4)	2.759 (3)	167 (4)
C2—H2*B*⋯S31	0.97	2.58	3.200 (2)	122
C6—H6*A*⋯S51	0.97	2.53	3.208 (2)	127
C13—H13⋯O4	0.93	2.26	2.693 (3)	108
C15—H15⋯O4	0.93	2.28	2.711 (3)	108
C35—H35⋯O1*W*^ii^	0.93	2.34	3.222 (4)	159
C55—H55⋯O1*W*^iii^	0.93	2.52	3.450 (4)	176
C56—H56*B*⋯*Cg*1^i^	0.96	2.97	3.763 (3)	141
C36—H36*C*⋯*Cg*2^i^	0.96	2.83	3.742 (3)	159

Symmetry codes: (i) 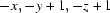; (ii) 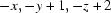; (iii) 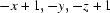. *Cg*1 and *Cg*2 are the centroids of the S31/C32–C35 and S51/C52–C55 rings ,respectively.

**Fig. 1.**
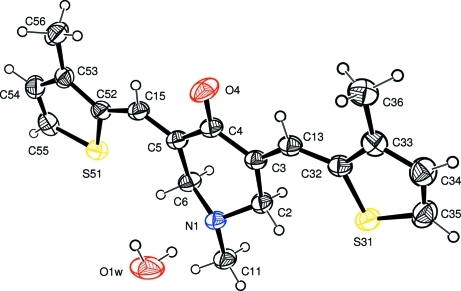
The molecular structure of (I), showing the atom-numbering scheme and displacement ellipsoids drawn at the 30% probability level. H atoms are shown as small spheres of arbitrary radius.

**Fig. 2.**
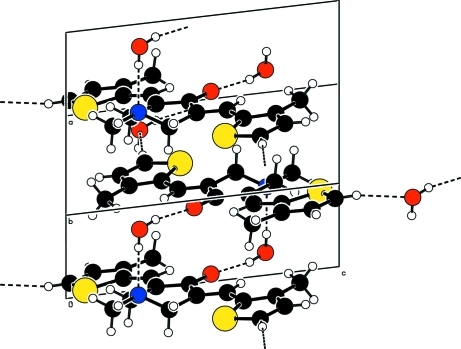
A view down the b axis of the packing in (I). Dashed lines indicate hydrogen bonds. The H atoms not involved in hydrogen bonding have been omitted for reasons of clarity.

## supplementary crystallographic information

### Comment

1-*N*-(Arylmaleamoyl)-3,5-bis(phenylmethylene)piperidin-4-ones (Jha & Dimmock, 2006) have been proved as antimycobacterial agents. The cytotoxic properties of 3,5 bis(arylidene)piperidin-4-ones (Das *et al.*, 2007) have also been reported. Due to the above importance, the crystal structure of the title compound (I) has been determined by X-ray diffraction.

The piperidine ring in (I), Fig. 1, adopts an envelope conformation with the methyl substituent in an equatorial position. The sum of the bond angles around N1 [330.9 (2)°] indicates a pyramidal geometry. The N1 atom deviates by -0.645 (3) Å from the least-squares plane passing through atoms C2—C6 . Both olefinic double bonds have an E-configuration. The thiophene rings are co-planar with the adjacent olefinic double bonds and the planar portion of piperidone ring. The dihedral angle between the two thiophene rings is 6.04 (14)°. The molecular conformation is stabilized by weak C—H···O and C—H···S contacts, Table 1. The water molecules forms two O—H donor interactions, one with the carbonyl-O atom and other to the amine-N atom, Table 1. These hydrogen bonds result in the formation of a centrosymmetric {C_18_H_19_NOS_2_.H_2_O}_2_ pair, and these are linked into supramolecular chains via C35-H···O contacts, Table 1. Additional stabilisation to the crystal strucutre is afforded by C-H···O and C-H···π contacts, as detailed in Table 1 and illustrated in Fig. 2.

### Experimental

To a mixture of *N*-methylpiperidin-4-one (1.5 ml, 0.01 mol) and 3-methylthiophene-2-aldehyde (2.7 ml, 0.02 mol) in ethanol (95%, 10 ml), sodium hydroxide (20%, 5 ml) was added. The solution was heated on a waterbath for 30 mins. The solid that separated on cooling was filtered and was recrystallized from 95% ethanol in a yield of 2.5 g (80%).

### Refinement

The water-H atoms, H1A and H1B, were located in a difference density Fourier map and included in the refinement with the O—H distances restrained to be 0.86±0.01 Å, with the H···H distance restrained to 1.373 Å, and with *U*_iso_(H) = 1.5 times *U*_eq_(O). The remaining H atoms were positioned geometrically and allowed to ride on their parent atoms with C—H = 0.93 - 0.97 Å, and with *U*_iso_(H) = 1.2 - 1.5 times *U*_eq_(C).

### Crystal data

**Table:**

C_18_H_19_NOS_2_·H_2_O	*Z* = 2
*M**_r_* = 347.50	*F*(000) = 368
Triclinic, *P*1	*D*_x_ = 1.290 Mg m^−^^3^
Hall symbol: -P 1	Melting point: 423 K
*a* = 7.5781 (7) Å	Mo *K*α radiation, λ = 0.71073 Å
*b* = 10.9926 (9) Å	Cell parameters from 9874 reflections
*c* = 11.5304 (10) Å	θ = 2.0–25.0°
α = 79.531 (2)°	µ = 0.31 mm^−^^1^
β = 83.404 (2)°	*T* = 293 K
γ = 71.673 (2)°	Prism, colourless
*V* = 894.90 (14) Å^3^	0.36 × 0.22 × 0.22 mm

### Data collection

**Table:**

Bruker Kappa APEXII CCD diffractometer	3127 independent reflections
Radiation source: fine-focus sealed tube	2686 reflections with *I* > 2σ(*I*)
graphite	*R*_int_ = 0.021
Detector resolution: 0 pixels mm^-1^	θ_max_ = 25.0°, θ_min_ = 2.0°
ω and φ scans	*h* = −9→9
Absorption correction: multi-scan (*SADABS*; Bruker, 2004)	*k* = −13→13
*T*_min_ = 0.901, *T*_max_ = 0.938	*l* = −13→13
15827 measured reflections	

### Refinement

**Table:**

Refinement on *F*^2^	Primary atom site location: structure-invariant direct methods
Least-squares matrix: full	Secondary atom site location: difference Fourier map
*R*[*F*^2^ > 2σ(*F*^2^)] = 0.043	Hydrogen site location: inferred from neighbouring sites
*wR*(*F*^2^) = 0.130	H atoms treated by a mixture of independent and constrained refinement
*S* = 1.11	*w* = 1/[σ^2^(*F*_o_^2^) + (0.0602*P*)^2^ + 0.4317*P*] where *P* = (*F*_o_^2^ + 2*F*_c_^2^)/3
3127 reflections	(Δ/σ)_max_ = 0.001
217 parameters	Δρ_max_ = 0.44 e Å^−^^3^
3 restraints	Δρ_min_ = −0.31 e Å^−^^3^

### Special details

**Table:**

Geometry. Bond distances, angles *etc*. have been calculated using the rounded fractional coordinates. All su's are estimated from the variances of the (full) variance-covariance matrix. The cell e.s.d.'s are taken into account in the estimation of distances, angles and torsion angles
Refinement. Refinement of *F*^2^ against ALL reflections. The weighted *R*-factor *wR* and goodness of fit *S* are based on *F*^2^, conventional *R*-factors *R* are based on *F*, with *F* set to zero for negative *F*^2^. The threshold expression of *F*^2^ > 2σ(*F*^2^) is used only for calculating *R*-factors(gt) *etc*. and is not relevant to the choice of reflections for refinement. *R*-factors based on *F*^2^ are statistically about twice as large as those based on *F*, and *R*- factors based on ALL data will be even larger.

### Fractional atomic coordinates and isotropic or equivalent isotropic displacement parameters (Å^2^)

**Table:**

	*x*	*y*	*z*	*U*_iso_*/*U*_eq_	
S31	0.12090 (9)	0.66376 (7)	0.92973 (5)	0.0616 (2)	
S51	0.60928 (9)	0.12751 (6)	0.41757 (6)	0.0613 (2)	
O4	0.1381 (3)	0.60702 (18)	0.46923 (16)	0.0789 (7)	
N1	0.3386 (3)	0.31766 (17)	0.73473 (15)	0.0491 (6)	
C2	0.3009 (4)	0.4473 (2)	0.7629 (2)	0.0561 (8)	
C3	0.1977 (3)	0.5506 (2)	0.67020 (19)	0.0481 (7)	
C4	0.2189 (3)	0.5242 (2)	0.54771 (19)	0.0517 (7)	
C5	0.3416 (3)	0.3973 (2)	0.52192 (19)	0.0491 (7)	
C6	0.4442 (3)	0.3036 (2)	0.6214 (2)	0.0562 (8)	
C11	0.4415 (4)	0.2222 (3)	0.8279 (2)	0.0644 (9)	
C13	0.0932 (3)	0.6700 (2)	0.6881 (2)	0.0496 (7)	
C15	0.3552 (3)	0.3766 (2)	0.40958 (19)	0.0503 (7)	
C32	0.0478 (3)	0.7334 (2)	0.7898 (2)	0.0508 (7)	
C33	−0.0590 (3)	0.8610 (2)	0.7893 (2)	0.0580 (8)	
C34	−0.0799 (4)	0.8984 (3)	0.9011 (3)	0.0717 (10)	
C35	0.0091 (4)	0.8029 (3)	0.9852 (3)	0.0747 (11)	
C36	−0.1438 (4)	0.9519 (3)	0.6826 (3)	0.0700 (9)	
C52	0.4595 (3)	0.2700 (2)	0.3507 (2)	0.0520 (8)	
C53	0.4559 (3)	0.2664 (3)	0.2315 (2)	0.0564 (8)	
C54	0.5707 (4)	0.1487 (3)	0.1991 (2)	0.0660 (9)	
C55	0.6615 (4)	0.0657 (3)	0.2886 (3)	0.0688 (10)	
C56	0.3477 (4)	0.3754 (3)	0.1436 (2)	0.0733 (10)	
O1W	0.0177 (4)	0.2338 (3)	0.7303 (2)	0.1048 (11)	
H2A	0.41793	0.46199	0.77200	0.0673*	
H2B	0.22827	0.45349	0.83775	0.0673*	
H6A	0.46857	0.21572	0.60610	0.0675*	
H6B	0.56308	0.31811	0.62516	0.0675*	
H11A	0.55653	0.23881	0.83494	0.0966*	
H11B	0.46732	0.13670	0.80858	0.0966*	
H11C	0.36829	0.22828	0.90153	0.0966*	
H13	0.03931	0.72188	0.62050	0.0595*	
H15	0.28175	0.44574	0.35991	0.0604*	
H34	−0.14837	0.98102	0.91613	0.0860*	
H35	0.00891	0.81227	1.06375	0.0897*	
H36A	−0.05985	0.93530	0.61429	0.1049*	
H36B	−0.16638	1.03985	0.69444	0.1049*	
H36C	−0.25932	0.93860	0.67070	0.1049*	
H54	0.58282	0.12987	0.12261	0.0792*	
H55	0.74340	−0.01605	0.28108	0.0825*	
H56A	0.26073	0.44002	0.18485	0.1096*	
H56B	0.28137	0.34220	0.09710	0.1096*	
H56C	0.43205	0.41357	0.09267	0.1096*	
H1A	0.099 (5)	0.273 (4)	0.734 (4)	0.1573*	
H1B	−0.046 (5)	0.280 (4)	0.672 (3)	0.1573*	

### Atomic displacement parameters (Å^2^)

**Table:**

	*U*^11^	*U*^22^	*U*^33^	*U*^12^	*U*^13^	*U*^23^
S31	0.0677 (4)	0.0680 (4)	0.0491 (4)	−0.0236 (3)	−0.0051 (3)	−0.0026 (3)
S51	0.0647 (4)	0.0559 (4)	0.0604 (4)	−0.0181 (3)	−0.0016 (3)	−0.0035 (3)
O4	0.0979 (14)	0.0685 (12)	0.0485 (10)	0.0049 (10)	−0.0227 (10)	0.0036 (9)
N1	0.0583 (11)	0.0452 (10)	0.0402 (9)	−0.0132 (8)	−0.0086 (8)	0.0021 (8)
C2	0.0684 (15)	0.0514 (13)	0.0466 (12)	−0.0150 (11)	−0.0143 (11)	−0.0016 (10)
C3	0.0477 (12)	0.0487 (12)	0.0466 (12)	−0.0157 (10)	−0.0068 (9)	0.0008 (9)
C4	0.0536 (13)	0.0521 (13)	0.0446 (12)	−0.0123 (10)	−0.0099 (10)	0.0025 (10)
C5	0.0491 (12)	0.0531 (12)	0.0436 (11)	−0.0169 (10)	−0.0050 (9)	0.0003 (9)
C6	0.0559 (14)	0.0571 (14)	0.0469 (12)	−0.0084 (11)	−0.0046 (10)	−0.0002 (10)
C11	0.0805 (18)	0.0536 (14)	0.0501 (13)	−0.0102 (12)	−0.0153 (12)	0.0041 (11)
C13	0.0463 (12)	0.0500 (12)	0.0493 (12)	−0.0143 (10)	−0.0070 (9)	0.0025 (10)
C15	0.0509 (12)	0.0545 (13)	0.0444 (12)	−0.0182 (10)	−0.0071 (9)	0.0021 (10)
C32	0.0451 (12)	0.0544 (13)	0.0522 (13)	−0.0176 (10)	−0.0021 (10)	−0.0021 (10)
C33	0.0458 (12)	0.0588 (14)	0.0672 (15)	−0.0147 (10)	0.0035 (11)	−0.0106 (12)
C34	0.0656 (16)	0.0710 (17)	0.0754 (18)	−0.0145 (14)	0.0074 (14)	−0.0215 (14)
C35	0.0772 (19)	0.093 (2)	0.0607 (16)	−0.0330 (16)	0.0111 (14)	−0.0255 (15)
C36	0.0591 (15)	0.0571 (15)	0.0807 (18)	−0.0026 (12)	−0.0072 (13)	−0.0027 (13)
C52	0.0513 (13)	0.0602 (14)	0.0497 (12)	−0.0272 (11)	−0.0026 (10)	−0.0036 (10)
C53	0.0563 (14)	0.0712 (15)	0.0519 (13)	−0.0328 (12)	−0.0003 (10)	−0.0129 (11)
C54	0.0687 (16)	0.0806 (18)	0.0608 (15)	−0.0361 (14)	0.0044 (13)	−0.0224 (14)
C55	0.0650 (16)	0.0635 (16)	0.0848 (19)	−0.0271 (13)	0.0106 (14)	−0.0246 (14)
C56	0.0785 (18)	0.099 (2)	0.0501 (14)	−0.0375 (16)	−0.0123 (13)	−0.0066 (14)
O1W	0.114 (2)	0.126 (2)	0.0823 (15)	−0.0674 (16)	−0.0458 (14)	0.0452 (14)

### Geometric parameters (Å, °)

**Table:**

S31—C32	1.728 (2)	C53—C56	1.506 (4)
S31—C35	1.698 (3)	C53—C54	1.401 (4)
S51—C52	1.726 (2)	C54—C55	1.341 (4)
S51—C55	1.701 (3)	C2—H2A	0.9700
O4—C4	1.225 (3)	C2—H2B	0.9700
O1W—H1A	0.86 (4)	C6—H6B	0.9700
O1W—H1B	0.86 (4)	C6—H6A	0.9700
N1—C11	1.460 (3)	C11—H11C	0.9600
N1—C2	1.452 (3)	C11—H11B	0.9600
N1—C6	1.459 (3)	C11—H11A	0.9600
C2—C3	1.495 (3)	C13—H13	0.9300
C3—C4	1.474 (3)	C15—H15	0.9300
C3—C13	1.342 (3)	C34—H34	0.9300
C4—C5	1.473 (3)	C35—H35	0.9300
C5—C15	1.342 (3)	C36—H36A	0.9600
C5—C6	1.501 (3)	C36—H36C	0.9600
C13—C32	1.426 (3)	C36—H36B	0.9600
C15—C52	1.429 (3)	C54—H54	0.9300
C32—C33	1.381 (3)	C55—H55	0.9300
C33—C34	1.400 (4)	C56—H56C	0.9600
C33—C36	1.502 (4)	C56—H56A	0.9600
C34—C35	1.354 (5)	C56—H56B	0.9600
C52—C53	1.386 (3)		
			
S31···C2	3.200 (2)	C54···H36C^iii^	2.9700
S31···C54^i^	3.611 (3)	C55···H36C^iii^	3.0400
S51···C6	3.208 (2)	C56···H15	2.6900
S51···C13^i^	3.614 (2)	H1A···N1	2.03 (4)
S51···C36^ii^	3.669 (3)	H1A···C2	2.88 (4)
S31···H56B^iii^	3.1200	H1A···H35^iv^	2.5000
S31···H2B	2.5800	H1A···C11	2.78 (4)
S51···H6A	2.5300	H1A···C6	2.87 (4)
O1W···N1	2.867 (4)	H1B···O4^iii^	1.91 (4)
O1W···O4^iii^	2.759 (3)	H2A···H11A	2.3600
O1W···C35^iv^	3.222 (4)	H2A···H6B	2.4600
O4···O1W^iii^	2.759 (3)	H2B···C32	2.9200
O1W···H55^v^	2.5200	H2B···H11C	2.3800
O1W···H35^iv^	2.3400	H2B···S31	2.5800
O4···H15	2.2800	H6A···S51	2.5300
O4···H13	2.2600	H6A···H11B	2.3400
O4···H6B^i^	2.7000	H6A···C52	2.9000
O4···H1B^iii^	1.91 (4)	H6B···H11A	2.4200
N1···O1W	2.867 (4)	H6B···H2A	2.4600
N1···H1A	2.03 (4)	H6B···O4^i^	2.7000
C2···S31	3.200 (2)	H6B···C4^i^	3.0800
C6···S51	3.208 (2)	H11A···H2A	2.3600
C13···S51^i^	3.614 (2)	H11A···H6B	2.4200
C32···C55^i^	3.525 (4)	H11B···H6A	2.3400
C32···C56^iii^	3.521 (4)	H11C···H2B	2.3800
C32···C54^i^	3.543 (4)	H11C···C35^iv^	3.1000
C33···C55^i^	3.342 (4)	H13···C36	2.6600
C35···O1W^iv^	3.222 (4)	H13···H36A	2.2200
C36···S51^vi^	3.669 (3)	H13···O4	2.2600
C54···C32^i^	3.543 (4)	H15···O4	2.2800
C54···S31^i^	3.611 (3)	H15···C56	2.6900
C55···C32^i^	3.525 (4)	H15···H56A	2.0600
C55···C33^i^	3.342 (4)	H34···H36B	2.5300
C56···C32^iii^	3.521 (4)	H35···H1A^iv^	2.5000
C2···H1A	2.88 (4)	H35···O1W^iv^	2.3400
C4···H6B^i^	3.0800	H36A···C13	2.7900
C6···H1A	2.87 (4)	H36A···H13	2.2200
C11···H1A	2.78 (4)	H36B···H34	2.5300
C13···H36A	2.7900	H36C···C55^iii^	3.0400
C15···H56A	2.6800	H36C···C53^iii^	3.0600
C32···H2B	2.9200	H36C···C54^iii^	2.9700
C32···H56B^iii^	2.9600	H55···O1W^v^	2.5200
C35···H11C^iv^	3.1000	H56A···H15	2.0600
C36···H13	2.6600	H56A···C15	2.6800
C52···H6A	2.9000	H56B···S31^iii^	3.1200
C53···H36C^iii^	3.0600	H56B···C32^iii^	2.9600
			
C32—S31—C35	92.06 (14)	H2A—C2—H2B	108.00
C52—S51—C55	91.93 (14)	C3—C2—H2A	109.00
H1A—O1W—H1B	104 (4)	N1—C6—H6A	109.00
C2—N1—C6	111.48 (18)	C5—C6—H6A	109.00
C2—N1—C11	109.72 (19)	C5—C6—H6B	109.00
C6—N1—C11	109.73 (19)	N1—C6—H6B	109.00
N1—C2—C3	112.71 (19)	H6A—C6—H6B	108.00
C2—C3—C4	118.61 (19)	N1—C11—H11B	109.00
C2—C3—C13	125.0 (2)	N1—C11—H11C	109.00
C4—C3—C13	116.3 (2)	H11A—C11—H11B	109.00
O4—C4—C3	120.4 (2)	H11A—C11—H11C	109.00
O4—C4—C5	121.1 (2)	H11B—C11—H11C	109.00
C3—C4—C5	118.50 (19)	N1—C11—H11A	109.00
C4—C5—C15	116.6 (2)	C3—C13—H13	113.00
C6—C5—C15	125.4 (2)	C32—C13—H13	114.00
C4—C5—C6	117.98 (18)	C52—C15—H15	113.00
N1—C6—C5	111.81 (19)	C5—C15—H15	113.00
C3—C13—C32	133.0 (2)	C33—C34—H34	123.00
C5—C15—C52	133.1 (2)	C35—C34—H34	123.00
S31—C32—C33	110.37 (17)	C34—C35—H35	124.00
C13—C32—C33	124.3 (2)	S31—C35—H35	124.00
S31—C32—C13	125.32 (17)	C33—C36—H36A	109.00
C32—C33—C36	125.1 (2)	C33—C36—H36C	109.00
C34—C33—C36	122.7 (2)	H36A—C36—H36B	109.00
C32—C33—C34	112.2 (2)	H36A—C36—H36C	109.00
C33—C34—C35	113.6 (3)	H36B—C36—H36C	109.00
S31—C35—C34	111.7 (3)	C33—C36—H36B	110.00
S51—C52—C15	124.92 (17)	C55—C54—H54	123.00
S51—C52—C53	110.18 (18)	C53—C54—H54	123.00
C15—C52—C53	124.9 (2)	S51—C55—H55	124.00
C52—C53—C56	125.4 (3)	C54—C55—H55	124.00
C54—C53—C56	122.5 (2)	C53—C56—H56B	109.00
C52—C53—C54	112.1 (2)	C53—C56—H56C	109.00
C53—C54—C55	113.8 (2)	C53—C56—H56A	110.00
S51—C55—C54	112.0 (2)	H56A—C56—H56C	109.00
N1—C2—H2A	109.00	H56B—C56—H56C	109.00
N1—C2—H2B	109.00	H56A—C56—H56B	109.00
C3—C2—H2B	109.00		
			
C35—S31—C32—C13	179.2 (2)	C4—C5—C6—N1	31.7 (3)
C35—S31—C32—C33	0.3 (2)	C15—C5—C6—N1	−150.0 (2)
C32—S31—C35—C34	−0.1 (3)	C4—C5—C15—C52	178.7 (2)
C55—S51—C52—C15	179.7 (2)	C6—C5—C15—C52	0.3 (4)
C55—S51—C52—C53	−0.7 (2)	C3—C13—C32—S31	−1.2 (4)
C52—S51—C55—C54	0.2 (3)	C3—C13—C32—C33	177.5 (3)
C6—N1—C2—C3	57.5 (3)	C5—C15—C52—S51	−0.2 (4)
C11—N1—C2—C3	179.2 (2)	C5—C15—C52—C53	−179.8 (3)
C2—N1—C6—C5	−59.9 (3)	S31—C32—C33—C34	−0.5 (3)
C11—N1—C6—C5	178.4 (2)	S31—C32—C33—C36	179.2 (2)
N1—C2—C3—C4	−26.9 (3)	C13—C32—C33—C34	−179.4 (2)
N1—C2—C3—C13	156.9 (2)	C13—C32—C33—C36	0.2 (4)
C2—C3—C4—O4	−179.2 (2)	C32—C33—C34—C35	0.4 (4)
C2—C3—C4—C5	−0.3 (3)	C36—C33—C34—C35	−179.2 (3)
C13—C3—C4—O4	−2.7 (4)	C33—C34—C35—S31	−0.2 (4)
C13—C3—C4—C5	176.3 (2)	S51—C52—C53—C54	1.1 (3)
C2—C3—C13—C32	−0.3 (4)	S51—C52—C53—C56	−177.2 (2)
C4—C3—C13—C32	−176.6 (2)	C15—C52—C53—C54	−179.3 (2)
O4—C4—C5—C6	176.6 (2)	C15—C52—C53—C56	2.4 (4)
O4—C4—C5—C15	−1.9 (4)	C52—C53—C54—C55	−1.0 (4)
C3—C4—C5—C6	−2.3 (3)	C56—C53—C54—C55	177.4 (3)
C3—C4—C5—C15	179.2 (2)	C53—C54—C55—S51	0.4 (4)

Symmetry codes: (i) −*x*+1, −*y*+1, −*z*+1; (ii) *x*+1, *y*−1, *z*; (iii) −*x*, −*y*+1, −*z*+1; (iv) −*x*, −*y*+1, −*z*+2; (v) −*x*+1, −*y*, −*z*+1; (vi) *x*−1, *y*+1, *z*.

### Hydrogen-bond geometry (Å, °)

**Table:**

*D*—H···*A*	*D*—H	H···*A*	*D*···*A*	*D*—H···*A*
O1W—H1A···N1	0.86 (4)	2.03 (4)	2.867 (4)	164 (4)
O1W—H1B···O4^iii^	0.86 (4)	1.91 (4)	2.759 (3)	167 (4)
C2—H2B···S31	0.97	2.58	3.200 (2)	122
C6—H6A···S51	0.97	2.53	3.208 (2)	127
C13—H13···O4	0.93	2.26	2.693 (3)	108
C15—H15···O4	0.93	2.28	2.711 (3)	108
C35—H35···O1W^iv^	0.93	2.34	3.222 (4)	159
C55—H55···O1W^v^	0.93	2.52	3.450 (4)	176
C56—H56B···Cg(1)^iii^	0.96	2.97	3.763 (3)	141
C36—H36C···Cg(2)^iii^	0.96	2.83	3.742 (3)	159

Symmetry codes: (iii) −*x*, −*y*+1, −*z*+1; (iv) −*x*, −*y*+1, −*z*+2; (v) −*x*+1, −*y*, −*z*+1.
